# Immunosuppressive Treg cells acquire the phenotype of effector-T cells in chronic lymphocytic leukemia patients

**DOI:** 10.1186/s12967-018-1545-0

**Published:** 2018-06-20

**Authors:** Serena De Matteis, Chiara Molinari, Giulia Abbati, Tania Rossi, Roberta Napolitano, Martina Ghetti, Andrea Ghelli Luserna Di Rorà, Gerardo Musuraca, Alessandro Lucchesi, Gian Matteo Rigolin, Antonio Cuneo, Daniele Calistri, Pier Paolo Fattori, Massimiliano Bonafè, Giovanni Martinelli

**Affiliations:** 1 Biosciences Laboratory, Istituto Scientifico Romagnolo per lo Studio e la Cura dei Tumori (IRST) IRCCS, via Maroncelli 40, 47014 Meldola, Italy; 2 Institute of Hematology “L. e A. Seragnoli”, Department of Experimental, Diagnostic and Specialty Medicine, University of Bologna, Bologna, Italy; 3 Hematology Unit, Istituto Scientifico Romagnolo per lo Studio e la Cura dei Tumori (IRST) IRCCS, Meldola, Italy; 4 Department of Medical Sciences, University of Ferrara-Arcispedale Sant’Anna, Ferrara, Italy; 5 Department of Experimental, Diagnostic & Specialty Medicine, Alma Mater Studiorum, University of Bologna, Bologna, Italy; 6 Scientific Directorate, Istituto Scientifico Romagnolo per lo Studio e la Cura dei Tumori (IRST) IRCCS, Meldola, Italy

**Keywords:** CLL, Tregs, Plasticity, Effector-like Tregs

## Abstract

**Background:**

In chronic lymphocytic leukemia (CLL) disease onset and progression are influenced by the behavior of specific CD4^+^ T cell subsets, such as T regulatory cells (Tregs). Here, we focused on the phenotypic and functional characterization of Tregs in CLL patients to improve our understanding of the putative mechanism by which these cells combine immunosuppressive and effector-like properties.

**Methods:**

Peripheral blood mononuclear cells were isolated from newly diagnosed CLL patients (n = 25) and healthy volunteers (n = 25). The phenotypic and functional characterization of Tregs and their subsets was assessed by flow cytometry. In vitro analysis of TH1, TH2, TH17 and Tregs cytokines was evaluated by IFN-γ, IL-4, IL-17A and IL-10 secretion assays. The transcriptional profiling of 84 genes panel was evaluated by RT^2^ Profiler PCR Array. Statistical analysis was carried out using exact non parametric Mann–Whitney U test.

**Results:**

In all CLL samples, we found a significant increase in the frequency of IL-10-secreting Tregs and Tregs subsets, a significant rise of TH2 IL-4^+^ and TH17 IL-17A^+^ cells, and a higher percentage of IFN-γ/IL-10 and IL-4/IL-10 double-releasing CD4^+^ T cells. In addition, we also observed the up-regulation of innate immunity genes and the down-regulation of adaptive immunity ones.

**Conclusions:**

Our data show that Tregs switch towards an effector-like phenotype in CLL patients. This multifaceted behavior is accompanied by an altered cytokine profiling and transcriptional program of immune genes, leading to a dysfunction in immune response in the peripheral blood environment of CLL patients.

**Electronic supplementary material:**

The online version of this article (10.1186/s12967-018-1545-0) contains supplementary material, which is available to authorized users.

## Background

Chronic lymphocytic leukemia (CLL) represents the most frequent leukemia in the Western world, with an annual incidence of about 4.5 new cases per 100,000 inhabitants [1]. CLL is characterized by clonal expansion and accumulation of mature CD5 positive B cells in the peripheral blood, bone marrow and secondary lymphoid organs [2]. Despite the introduction of promising therapeutic strategies, this disease remains incurable [3, 4]. T cell abnormalities are a peculiarity of CLL from the early stages onwards, regardless of disease progression [5]. The dysfunction of the innate and adaptive immune system also contribute to increased susceptibility to bacterial and opportunistic infections in CLL patients [6, 7].

Disease onset and progression are influenced by the behavior of specific CD4^+^ T cell subsets, such as T regulatory cells (Tregs) [8, 9], which are characterized by constitutive expression of high levels of the interleukin (IL)-2 receptor α chain (CD25). The majority of CD4^+^CD25^high^ Tregs also express a forkhead family transcription factor (FoxP3) which is required for both their differentiation and their immunosuppressive function [10]. The suppressive function of Tregs may be related to different factors, such as modulation of target cell signaling via cell–cell contact and/or secretion of immunosuppressive cytokines such as IL-10, IL-35 and transforming growth factor β (TGF-β) [11, 12].

Tregs are capable of trans-differentiating into other effector CD4^+^ T cell subsets, by undertaking T-helper specific transcriptional program and releasing cytokines related to effector-like T cells [13–15]. Currently, there is no definitive evidence of the involvement of these populations in CLL onset or progression.

Here, we focused our attention on the phenotypic and functional characterization of Tregs subsets in untreated CLL patients to improve our understanding about the putative mechanism, throughout which these cells combine immunosuppressive and effector-like properties.

## Methods

### Patient sample

The study was performed in accordance with the principles laid down in the Declaration of Helsinki. After obtaining patient informed consent and the approval of the local Ethics Committee (Prot. No. IRST B031; approval date: 21 January 2015), samples of peripheral blood (15–20 ml) were collected from 25 untreated CLL patients enrolled at Istituto Scientifico Romagnolo per lo Studio e la Cura dei Tumori (IRST) IRCCS in Meldola and at the Department of Medical Sciences of University of Ferrara—Arcispedale Sant’Anna in Ferrara. Another 25 healthy volunteers (HVs) were used as control. The need for donor consent was waived by the Ethics Committee. Clinical characteristics of patients are reported in Table 1.

**Table 1:** Clinical characteristics of CLL patients

	HV (n = 25)	Patients (n = 25)
Gender		
Male	12	12
Female	13	13
Median age, years (range)	63 (48–87)	73 (58–87)
RAI staging		
0–I		18
II		3
III		2
IV		2
Binet staging		
A/B/C		21/3/1
Karyotype		
Normal		20
Del(13q14)		3
Del(11q22)		1
Del(17p13)		1

*HV* healthy volunteers, *CLL* chronic lymphocytic leukemia

### Cell isolation and in vitro stimulation

Blood samples were collected in sterile EDTA tubes and peripheral blood mononuclear cells (PBMCs) were separated by density gradient centrifugation using Lymphosep (Biowest) and frozen in 90% heat-inactivated fetal bovine serum (FBS) (PAA) and 10% dimethylsulfoxide (Sigma Aldrich). In order to avoid contamination by CD4^+^ monocytes, these latter were depleted by CD14 MicroBeads-based negative selection (Miltenyi Biotec). Human CD4^+^ T cells were isolated by negative depletion of CD8^+^, CD14^+^, CD15^+^, CD16^+^, CD19^+^, CD36^+^, CD56^+^, CD123^+^, TCR y/δ and CD235a^+^ cells, using the CD4^+^ T cell isolation kit (Miltenyi Biotec), according to the manufacturer’s protocol. The isolated cells were fluorescently stained with CD4-FITC and analyzed by flow cytometry to verify the purity. Cells were cultured in RPMI 1640 medium (PAA) supplemented with 10% heat inactivated FBS, l-glutamine (2 mM, Euroclone), penicillin (100 U/ml) and streptomycin (100 μg/ml) (PAA). CD4^+^ cells were primed for 24 h at 37 °C with IL-6 (30 ng/ml, Miltenyi Biotec) overnight (o/n) and then incubated for 5 h at 37 °C with phorbol 12-myristate-13-acetate (P) (50 ng/ml), ionomycin (I) (1 μg/ml, Invitrogen) and GolgiStop Protein Transport Inhibitor (Monensin, BD recommended concentration) (M) based on polarization method previously reported by Musuraca et al. [16]. An unstimulated control, prepared by incubating CD4^+^ cells with GolgiStop Protein Transport Inhibitor, was included for each experiment.

### Tregs immunophenotypic analysis

For Tregs and effector-like T cells analysis, stimulated PBMCs were stained with CD4-FITC (0.6 μg/ml, clone SK3, BD Biosciences) and CD25-APC-Cy7 (2.5 μg/ml, clone M-A251, BD Biosciences) for 10 min at 4 °C in the dark. After incubation, cells were fixed, permeabilized and stained with FoxP3-APC (clone 3G3, Miltenyi Biotec) and with either Tbet-PE (clone REA102, Miltenyi Biotec) or GATA-3-PE (clone REA174, Miltenyi Biotec) or RORγt-PE (clone REA278, Miltenyi Biotec) for 30 min at 4 °C in the dark. Appropriate isotype controls were included for each sample.

### Cytokine secretion analysis

Stimulated CD4^+^ cells were washed with cold PBS containing 0.5% (v/v) bovine serum albumin (BSA) (Sigma Aldrich) and 2 mM of EDTA and analyzed using human IFN-γ, IL-4, IL-17A and IL-10 secretion assay—detection kits (Miltenyi Biotec) according to the manufacturer’s instructions. Samples were washed and suspended for flow cytometric analysis.

### T cell activation with *C. Albicans* and isolation of IL-17-secreting cells

CD4^+^ cells (2.5 × 10^6^) were stimulated for 24 h at 37 °C with 1 μg/ml of *C. albicans* peptides (JPT, Berlin, Germany). During the last 5 h of incubation, cells were maintained in the presence of GolgiStop Protein Transport Inhibitor (BD Pharmingen). Cells were fixed, permeabilized and stained with IFN-γ-FITC (Miltenyi Biotec). A sample stimulated with *C. Albicans* for 48 h without depletion of IL-17-secreting cells was added as control.

### Flow cytometry

Flow cytometric analysis was performed using a FACSCanto flow cytometer (Becton–Dickinson) equipped with 488 nm (blue) and 633 (red) lasers. 30.000 events were recorded for each sample. Acquisition and analysis gates were set on lymphocytes based on forward (FSC) and side scatter (SSC) properties of cells. FSC and SSC were set in a linear scale. Cell debris and dead cells were excluded from the analysis based on scatter signals and propidium iodide fluorescence. Flow cytometry data were analyzed with Diva Software (Becton–Dickinson).

### ELISA analysis

IL-23 levels were evaluated in plasma obtained from HV and CLL patients by ELISA kit (U-CyTech Biosciences) according to the manufacturer’s instructions.

### RNA extraction and RT^2^ Profiler PCR Arrays

Total RNA from 3 × 10^6^ of CD4^+^ T cells was isolated using miRNeasy Micro Kit (Qiagen) according to the manufacturer’s instructions. RNA concentration and quality were evaluated by Nanodrop-ND-1000 (Celbio). cDNA was synthesized from 250 ng of total RNA using RT^2^ First Strand Kit (SABiosciences Corp.) following the manufacturer’s instructions and used to analyze the expression levels of 84 genes (Additional file 2: Table S1) by RT^2^ Profiler Human Innate & Adaptive Immune Responses PCR Array (PAHS-052Z, SABiosciences Corp.). Real-Time PCR amplification was carried out on 7500 Real-Time PCR System (Applied Biosystems). The online tool RT^2^ Profiler data analysis software (Qiagen) was used for data normalization and statistical analyses. The threshold cut-off point was established at > 2.5-fold differential expression.

### Statistical analysis

Statistical analysis was carried out using exact nonparametric Mann–Whitney U test (GraphPad Prism 6), and data were summarized by the median and interquartile range. *P* values < 0.05 were considered as significant.

## Results

### Increased Tregs frequency in peripheral blood of untreated CLL patients

**Fig. 1:**
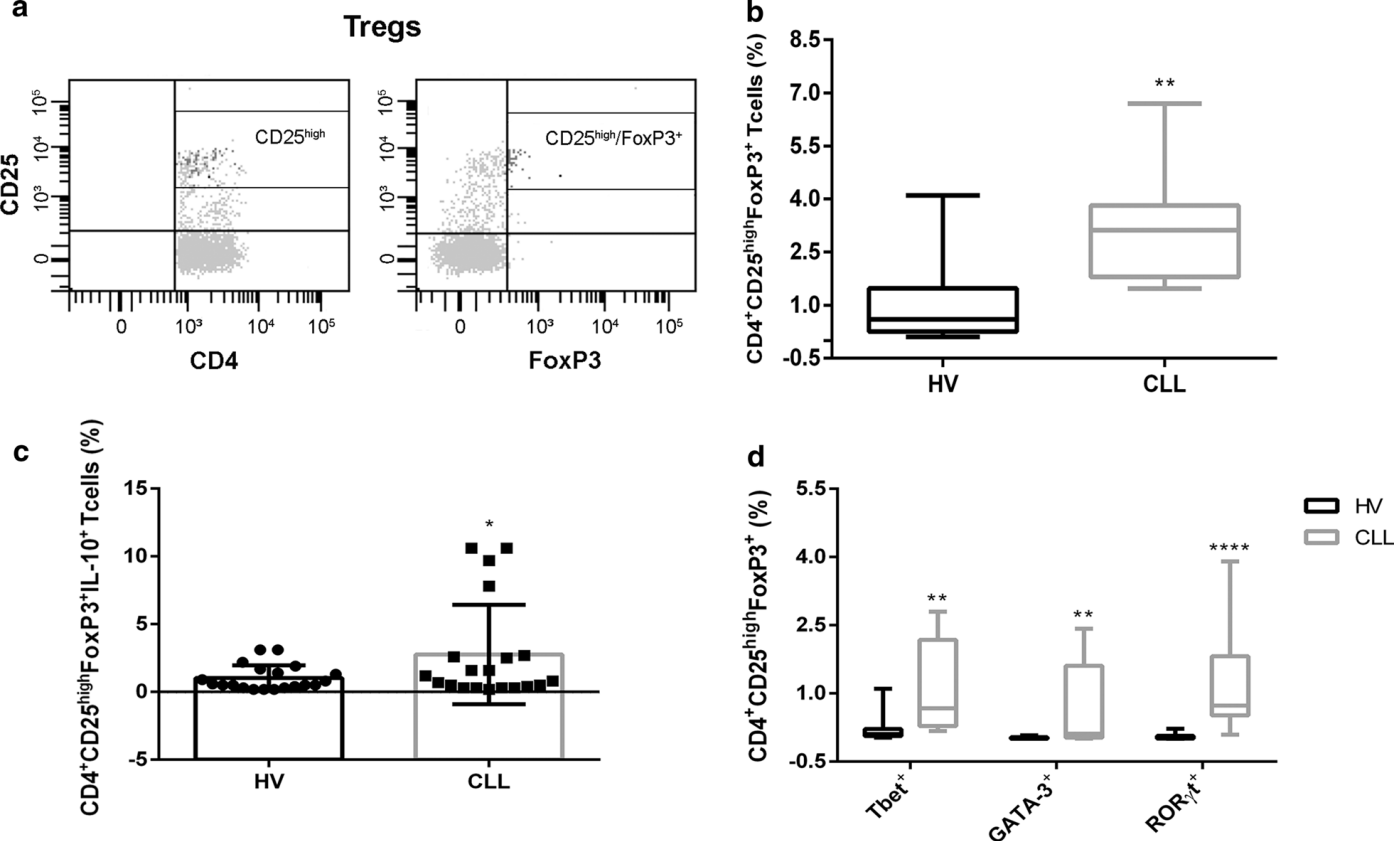
Increased Tregs frequency in peripheral blood of CLL patients. **a** Gating strategy used to identify Tregs as CD4^+^CD25^high^FoxP3^+^. Representative box plots of **b** Tregs frequency in PBMCs obtained from HV (n = 15) and CLL patients (n = 15); **c** IL-10-secreting Tregs frequency in PBMCs obtained from HV (n = 15) and CLL patients (n = 15); **d** Tregs subsets frequency in PBMCs obtained from HV (n = 15) and CLL patients (n = 15), all after in vitro priming with IL-6 and phorbol 12-myristate 13-acetate (P), ionomycin (I) and monensin (M). All results are expressed as median and interquartile range. P value shown is obtained from the comparison between the indicated groups by exact non-parametric Mann–Whitney U test (*P < 0.05; **P < 0.01; ***P < 0.001)

First, we focused on the frequency of circulating Tregs by assessing the percentage of CD4^+^CD25^high^FoxP3^+^ T cells in the peripheral blood of untreated CLL patients (n = 15) and sex-and age-matched HVs (n = 15). The flow cytometric analysis of Tregs was performed on PBMCs after priming with IL-6 o/n and PIM for 5 h. CD4^+^ T cells with a mean fluorescence intensity of CD25 expression ≥ 10-fold the negative cut-off were classified as CD25^high^ based on the data previously reported by Musuraca et al. [16] (Fig. 1a). A significantly higher frequency of Tregs was found in CLL patients compared to HVs in stimulated PBMCs (3.1%, range 1.5–6.7% vs 0.6%, range 0.1–4.1%, respectively, P = 0.006) (Fig. 1b). To further elucidate the suppressive role of Tregs, we evaluated their capacity to release IL-10, observing a significant increase of IL-10^+^ Tregs in patients with respect to HVs (1%, range 0.2–10.6% vs 0.6%, range 0.2–3.1%, respectively) (Fig. 1c).

### Tregs subsets expansion in the peripheral blood of untreated CLL patients

Owing to the Tregs capacity of transdifferentiation into other CD4^+^ T cells, we characterized the phenotypic profiling of Tregs subsets, by evaluating the co-expression of FoxP3 with effector-T-associated transcription factors, namely Tbet, GATA-3 and RORγt. We observed a significant increase in the frequency of all three Treg populations in CLL patients than HVs, namely Tregs Tbet^+^ (0.7%, range 0.2–2.8% vs 0.1%, range 0.03–1.1%, respectively), Tregs GATA-3^+^ (0.1%, range 0.01–2.4% vs 0%, range 0–0.1%, respectively), Tregs RORγt^+^ (0.7%, range 0.1–3.9% vs 0.03%, range 0–0.2%, respectively) (Fig. 1d). These results highlight the capacity of Tregs to switch in an effector-like phenotype.

### Alteration in T cell-associated cytokine profiling in the peripheral blood of untreated CLL patients

**Fig. 2:**
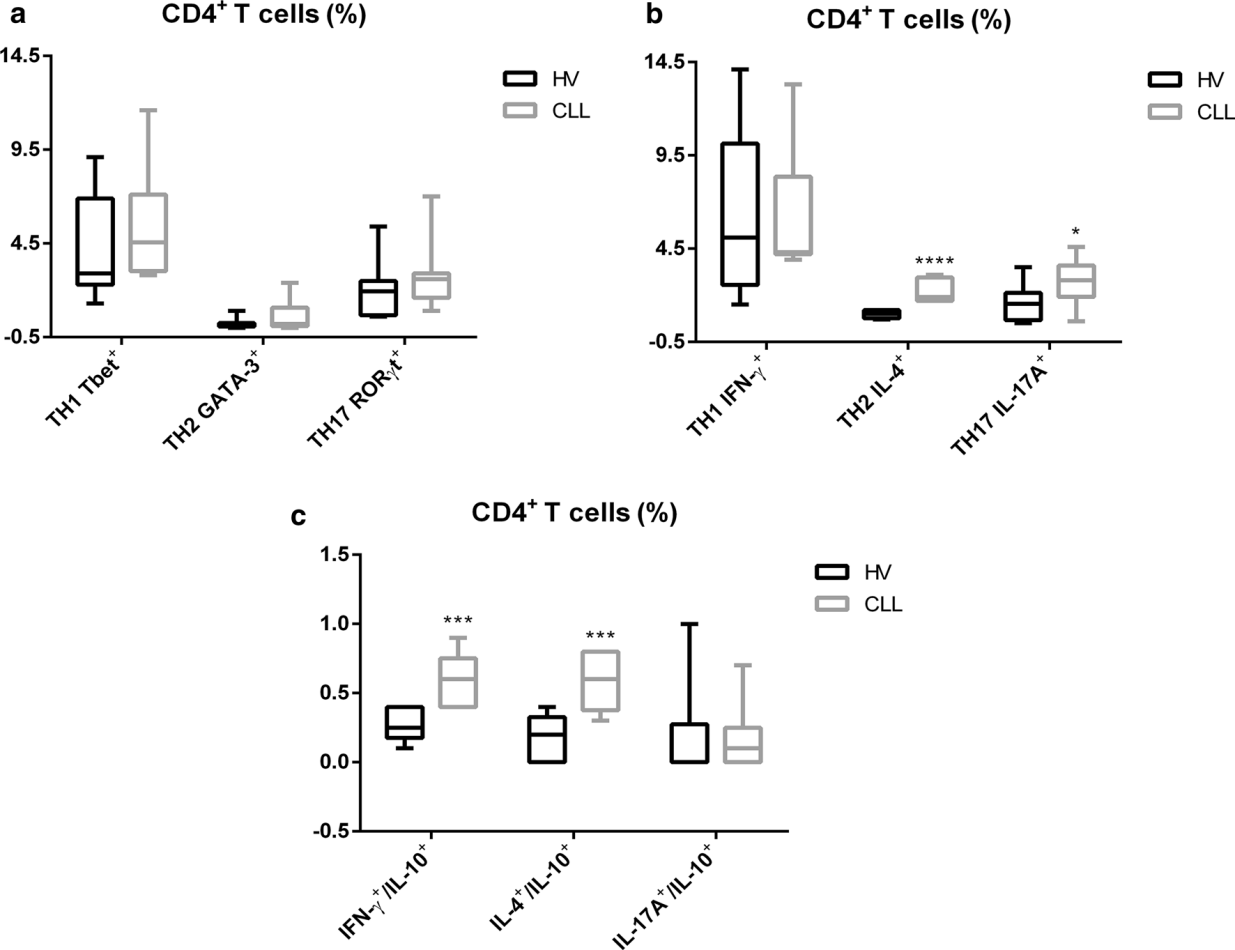
Evaluation of CD4^+^ T cells frequency in peripheral blood of CLL patients. Representative box plots relative to **a** CD4^+^ T cell subsets frequency in PBMCs from HV (n = 15) and CLL patients (n = 15); **b** IFN-γ, IL-4 and IL-17A secretion in CD4^+^ T cells obtained from HV (n = 15) and CLL patients (n = 15); **c** IFN-γ, IL-4, IL-17A co-secretion with IL-10 in CD4^+^ T cells obtained from HV (n = 15) and CLL patients (n = 15); all after in vitro stimulation with IL-6 and PIM. All results are expressed as median and interquartile range. P value shown is obtained from the comparison between the indicated groups by exact non-parametric Mann–Whitney U test (*P < 0.05; **P < 0.01; ***P < 0.001)

In addition to the analysis of the Tregs subpopulation, we characterized the phenotypic and functional profiling of TH1, TH2 and TH17 subsets in whole CD4^+^ T cells. First, we analysed the expression of Tbet, GATA-3 and RORγt in total CD4^+^ T cells after IL-6 o/n and PIM for 5 h, without observing any significant differences between patients and HVs (Fig. 2a). We then considered the capacity of CD4^+^ T subsets to release IFN-γ, IL-4, IL-17A after in vitro stimulation with IL-6 o/n and PIM for 5 h (Fig. 2b). A significant increase was seen in the frequency of TH2 IL-4^+^ and TH17 IL-17A^+^ cells in CLL patients with respect to HVs (1.9%, range 1.7–3.1% and 2.8%, range 0.6–4.6% vs 1%, range 0.7–1.2% and 1.6%, range 0.5–3.5%, respectively), whereas the percentage of TH1 IFN-γ^+^ cells remained unchanged (Fig. 2b). Moreover, we examined the ability of the in vitro stimulated CD4^+^ T cells to simultaneously secrete IFN-γ/IL-10, IL-4/IL-10 and IL-17A/IL-10 (Fig. 2c), thereby acquiring a Treg-like phenotype. We observed a statistically significant increase in the frequency of IFN-γ^+^/IL-10^+^ and IL-4^+^/IL-10^+^ cells in CLL patients compared to HVs (0.6%, range 0.4–0.9% and 0.6%, range 0.3–0.8% vs 0.25%, range 0.1–0.4% and 0.2%, range 0.0–0.4%, respectively), whereas the percentage of IL-17A^+^/IL-10^+^ cells remained unchanged (Fig. 2c). Owing to circulating Tregs that represent approximately 5–8% of total CD4^+^ T cells, the analysis could not be performed in this subset.

Overall, the altered cytokine profiling described in the peripheral blood environment in CLL patients, suggesting a dysfunction in immune response.

### Elevated IL-23 levels in the plasma of untreated CLL patients

The induction of the TH17-associated pro-inflammatory response is also likely to involve IL-23, which plays a key role in orchestrating T cell-mediated inflammatory pathways and promoting TH17 differentiation and function. We evaluated IL-23 level in plasma samples obtained from HV (n = 10) vs CLL (n = 10), observing a statistically significant increase in patients compared to HVs (57.21%, range 20.87–172.8% vs 13.3%, range 7.06–32.22%, respectively; P = 0.0003) (Additional file 1: Fig. S1). These results suggest that the increase of TH17 IL-17A^+^ observed in CLL patients may be attributed to the stimulatory interplay with IL-23.

### Increased frequency of IFN-γ^+^ CD4^+^ T cells after stimulation with *C. Albicans* in CLL patients

**Fig. 3:**
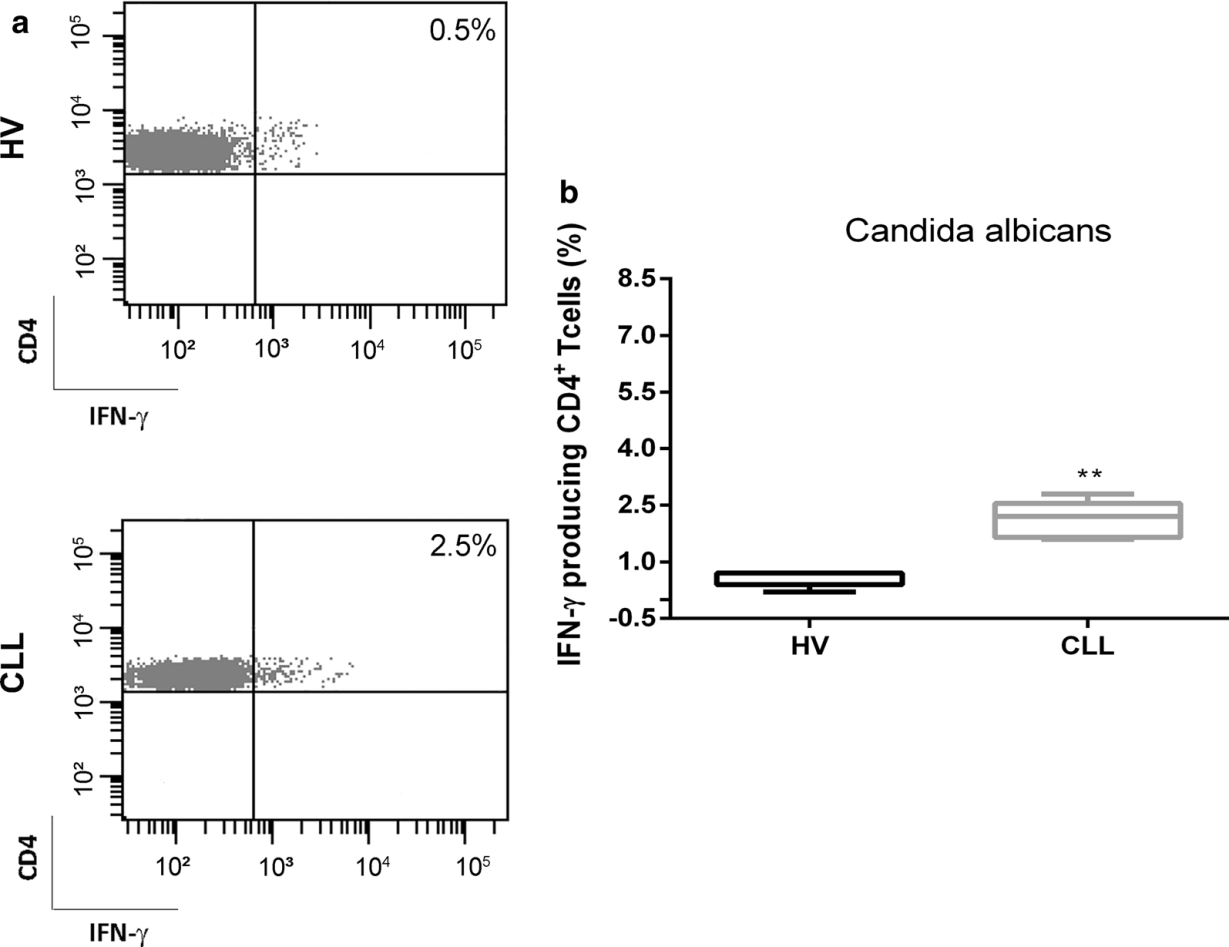
Increased frequency of IFN-γ^+^ CD4^+^ T cells after stimulation with *C. albicans* in CLL patients. **a** Representative dot plots of IFN-γ^+^ CD4^+^ T cells from a HV and a CLL patient are shown. **b** Representative box plots related to IFN-γ^+^ CD4^+^ T cell frequency in HV (N = 10) and CLL patients (N = 10). All results are expressed as median and interquartile range. P value shown is obtained from the comparison between the indicated groups by exact non-parametric Mann–Whitney U test (*P < 0.05)

In order to evaluate CD4^+^ cell-mediated immune response in terms of IFN-γ production, we in vitro stimulated CD4^+^ T cells with *C. Albicans* (Fig. 3a, b). As shown in Fig. 3b, the frequency of IFN-γ^+^ cells was higher in patients than in HVs (2.2%, range 1.6–2.8% vs 0.7%, range 0.2–0.7%, respectively; P = 0.008). This in vitro functional proof confirms that TH17 cells have a key role in promoting the inflammatory response not only by releasing large amount of IL-17, but also IFN-γ after fungal stimulus.

### Altered transcriptional profiling of genes involved in Innate and Adaptive Immune processes in CD4^+^ T cells of CLL patients

We evaluated the transcriptional profiling of 84 genes involved in Innate and Adaptive Immune processes in isolated CD4^+^ T cells from HVs (n = 10) and CLL patients (n = 10) after in vitro stimulation with IL-6 o/n and PIM for 5 h (Additional file 2: Table S1). We tried to counterbalance the limit of small number of samples by using a more stringent threshold to consider genes differentially expressed in a significant manner.

This panel was chosen because most of the genes studied were involved in development, differentiation and activity of total Tregs and subsets. The transcriptional profiling analysis was performed on total CD4^+^ T cells due to the limiting amount of total RNA from isolated circulating Tregs.

**Fig. 4:**
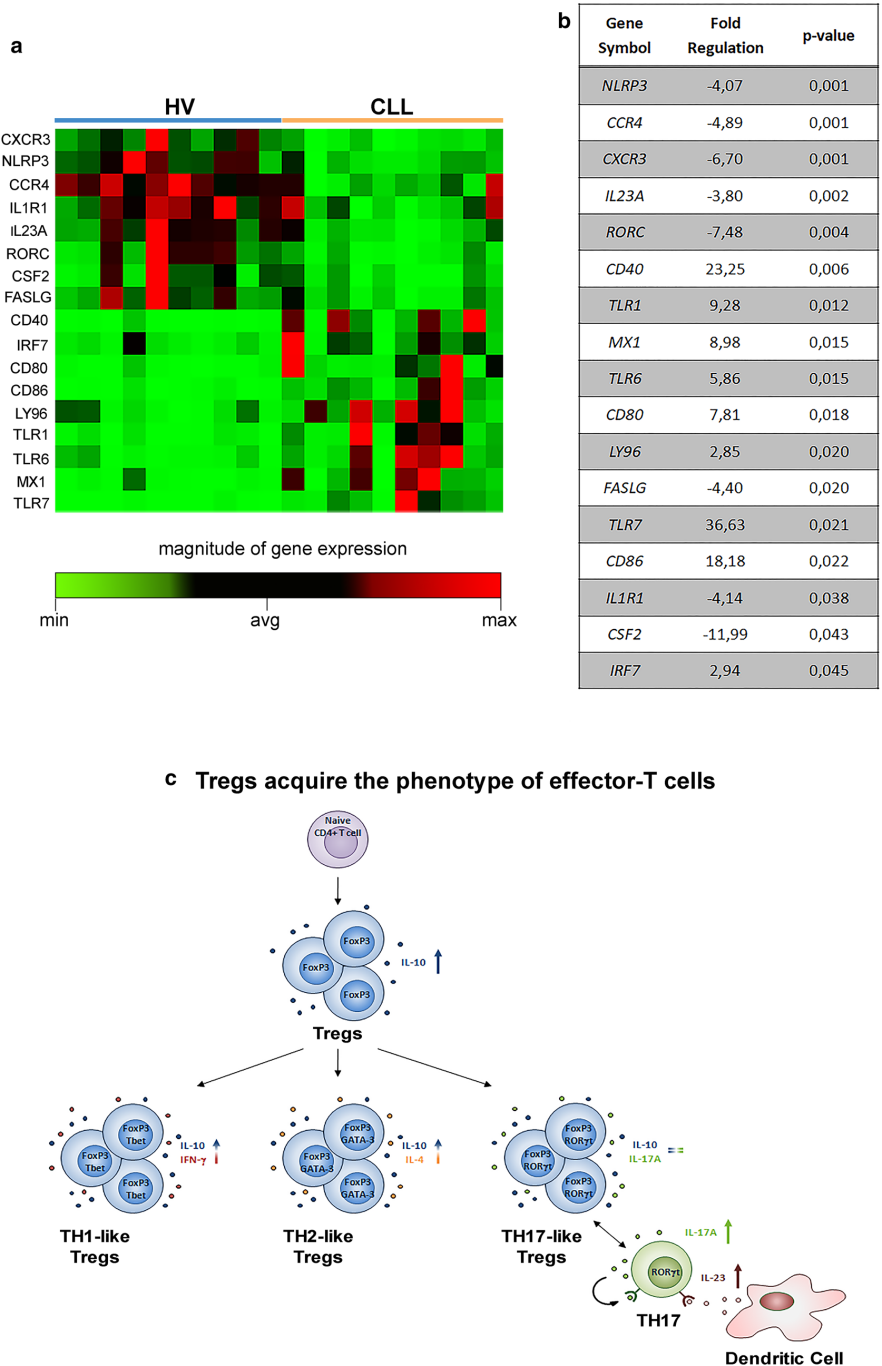
Innate and adaptive immune response-related gene abnormalities in CD4^+^ T cells of CLL patients. **a** Supervised clustering of differentially expressed genes in CD4^+^ T cells from HV (n = 10) and CLL patients (n = 10) (P < 0.05). **b** Fold regulation of significant differentially expressed genes. **c** Schematic representation of Tregs differentiation. Under differentiation stimuli, Tregs switched to effector-like Tregs, co-expressing FoxP3 and master transcription factors of effector-T cells and co-releasing IL-10 with inflammatory cytokines, such as IFN-γ and IL-4. However, Tregs did not show a TH17-like functional switch throughout the acquisition of RORγt transcription factor

A total of 17 (20%) genes were differentially expressed between CLL and HVs cells with changes > 2.5-fold (Fig. 4a, b). Among these, 9 genes (*CD40, IRF7, CD80, CD86, LY96, TLR1, TLR6, MX1 and TLR7*) were significantly up-regulated and 8 genes (*CXCR3, NLRP3, CCR4, IL1R1, IL23A, RORC, CSF2, FASLG*) resulted significantly down-regulated (Fig. 4a, b). The altered transcriptional profiling of CLL CD4^+^ T cells was characterized by the up-regulation of innate immunity and a down-regulation of adaptive immunity genes.

## Discussion

It has been reported that T cells abnormalities may contribute to the immunopathogenesis of CLL by supporting leukemic clone proliferation and survival [8, 9, 17]. Recent investigations on the role of Tregs have suggested that tumors may subvert tumor immunity by promoting the expansion, recruitment, and activation of this T cell subset [18, 19]. Interestingly, study by Ai et al. showed that follicular lymphoma B cells induced the phenotypic switch from effector-T cells into Tregs when co-cultured with PBMCs from healthy donors [20].

Our data describe a multifaceted identity of Tregs in CLL patients. In particular, Tregs show the capacity of switching towards an effector-like phenotype. In addition, we reported a dysfunction in immune response in the peripheral blood environment in CLL patients characterized by an altered cytokine and transcriptional profiling.

In line with several studies, we observed a significant increase in Tregs frequency and in their capacity to produce IL-10 in the peripheral blood of CLL patients with respect to HVs [21–30]. Our findings also revealed a higher expression of Tbet, GATA-3 and RORγt in FoxP3^+^Tregs in patients, suggesting a phenotypic switch towards TH1, TH2 and TH17 cells after differentiation stimuli (Fig. 4c). We also noted a significant increase in the capacity of CD4^+^ T cells to release IL-10 in combination with IFN-γ or IL-4, suggesting a Tregs-like behavior (Fig. 4c) [31]. On the one hand, Tregs combine immunosuppressive and inflammatory functions, on the other, TH17 and TH2 effector cells contribute to promote an inflammatory response to infectious stimuli. In line with the literature [32, 33], we showed an increased percentage of TH2 IL-4^+^ and TH17 IL-17A^+^ in CLL patients than HVs. In addition to these findings, we also described an altered transcriptional program in CLL patients. In particular, in patients we observed an up-regulation of members of the Toll-like receptor (TLR) family (*TLR1, TLR6* and *TLR7*) that seem to be involved in the modulation of Tregs suppressive function [34] and production of inflammatory cytokines [35]. Moreover, CD80 and CD86 involved in the generation of Tregs [18, 19], and CD40, a marker for pathogenetic T cells-producing pro-inflammatory cytokines such as IL-17A [36, 37], were present at high levels in CLL patients. Conversely, genes involved in apoptosis (*FASLG*) and leukocyte trafficking and differentiation (*CXCR3, CCR4, CSF2*), regulation of inflammatory response (*IL*-*1R1*) and inflammasome composition (*NLRP3*) were down-regulated. In particular, the low levels of *NLRP3* transcript in CLL patients may play a putative role in supporting leukemic cell growth, in line with data by Salaro et al. sustaining that NLRP3 interferes with CLL progression in virtue of its ability to modulate cytokine release and apoptosis, and thus controls inflammation [38]. With regard to the down-regulation of important genes involved in T cell development and differentiation, e.g. *CXCR3, IL*-*23A* and *RORC*, we hypothesize a mechanism of negative regulation related to a sub-subset of TH17 known as TH17-23 which generates the effector cells involved in anti-tumor immunity [39].

Our study has a number of limitations. First, the low number of enrolled patients and the short follow-up (1.5 years) did not allow us to correlate the biological values related to effector-like Tregs with clinical characteristics. Second, we performed some explorative analysis such as transcriptional profiling, Candida assay and IL-23 plasma levels on a small number of patients enrolled afterwards. As future perspectives, these analysis will be validated on a greater number of patients.

## Conclusions

Our cellular and molecular data highlighted, for the first time in CLL, the presence of specific subsets of Tregs that show phenotypic and functional characteristics of effector-T cells. We hypothesize that, on the one hand, these Tregs subsets may suppress adaptive immune responses, and on the other, create an inflammatory environment that probably sustains leukemic B clone survival and expansion. These subsets could thus represent an attractive and promising therapeutic target for the treatment of the disease thanks to their dual role.

## Additional files

**Additional file 1: Fig. S1.** Representative box plots relative to IL-23 levels (pg/ml) in plasma from HV (n = 10) and CLL (n = 10). Results are expressed as median and interquartile range. P value shown is obtained from the comparison between the indicated groups by exact non-parametric Mann–Withney U test (*P < 0.05).

**Additional file 2: Table S1.** List of genes involved in innate and adaptive immunity.

## Abbreviations

CLL: chronic lymphocytic leukemia
Tregs: T regulatory cells
CD25: interleukin (IL)-2 receptor α chain
FoxP3: forkhead family transcription factor
IL-10: interleukin-10
TGF-β: transforming growth factor β
eff-like: effector-like
HV: healthy volunteers
PBMCs: peripheral blood mononuclear cells
FBS: fetal bovine serum
BSA: bovine serum albumin
FSC: forward scatter
SSC: side scatter
TLR: Toll-like receptor
