# Supplementary material for: Immunosuppressive Treg cells acquire the phenotype of effector-T cells in chronic lymphocytic leukemia patients
Source: J Transl Med. 2018 Jun 20;16:172. doi: 10.1186/s12967-018-1545-0 (PMC6011245; doi:10.1186/s12967-018-1545-0)
Supplement: Supplementary file 1 — Additional file 1: Fig. S1. Representative box plots relative to IL-23 levels (pg/ml) in plasma from HV (n = 10) and CLL (n = 10). Results are expressed as median and interquartile range. P value shown is obtained from the comparison between the indicated groups by exact non-parametric Mann–Withney U test (*P < 0.05). [file 12967_2018_1545_MOESM1_ESM.docx]

**Additional file 1: Fig. S1**

Representative box plots relative to IL-23 levels (pg/ml) in plasma from HV (n = 10) and CLL (n = 10). Results are expressed as median and interquartile range. P value shown is obtained from the comparison between the indicated groups by exact non-parametric Mann-Withney U test (*P < 0.05).
